# A single-center experience of magnetic resonance imaging findings of fetal sacrococcygeal teratomas

**DOI:** 10.55730/1300-0144.5423

**Published:** 2022-05-07

**Authors:** Mesude TOSUN, İsa ÇAM, Hande USLU, Yasemin DOĞAN, Yonca ANIK

**Affiliations:** 1 Department of Radiology, Faculty of Medicine, Kocaeli University, Kocaeli, Turkey; 2 Department of Obstetrics and Gynecology, Faculty of Medicine, Kocaeli University, Kocaeli, Turkey

**Keywords:** Fetal, magnetic resonance imaging, sacrococcygeal teratoma, Altmand type, prenatal, diagnosis

## Abstract

**Background/aim:**

Sacrococcygeal teratomas (SCT) are known as rare tumors, but they are the most common tumor in fetuses and newborns. This study aims to present fetal magnetic resonance imaging (MRI) findings of SCT diagnosed prenatally and compare them with that of the prenatal Ultrasound (US) findings.

**Materials and methods:**

Eleven patients diagnosed as SCT prenatally by US and further assessed by MRI are included. MRI was performed via a 1.5-T magnet with a body coil. The presence, size, content extension, and compressive effects of each mass were determined and correlated with US findings and with postnatal examinations, including surgery and pathology. As complications, the presence of ascites and skin edema or pleural or pericardial effusion was diagnosed as hydrops. The amniotic fluid index was calculated for the assessment of oligo- or polyhydramnios.

**Results:**

US findings are found strongly correlated with MRI findings. An agreement on the extent of each mass was observed in eight patients, disagreement in one fetus was an extension of the tumor within the spinal canal recognized only at MR and assessment of intrapelvic-abdominal extension was superior in MRI. There were n = 6 type I, n = 2 type II, n = 1 type III and, n = 2 type IV tumors. MRI was superior to US for detecting displacement of the colon (n = 3), intrapelvic-intraabdominal extension (n = 1), urinary tract complication (n = 2), and intraspinal extension (n = 1).

**Conclusion:**

MRI is found to be superior to US especially in the assessment of intrapelvic and intraspinal extension of the tumor, colonic displacement, and complications.

## 1. Introduction

Sacrococcygeal teratomas (SCT) are known as rare tumors, but they are the most common tumor in fetuses and newborns. The incidence reported in the literature is 1/35,000 to 40,000 live births [1–3]. However, recent studies reported in the literature suggest that the incidence may be higher, and tumors diagnosed prenatally account for 50 of the cases [4,5]. The female to male ratio for SCT is reported to be 3:1 [6,7]. The vast majority of cases are sporadic. It has been defined as a neoplasm consisting of tissues from all three germ layers, or a neoplasm consisting of multiple foreign tissues with no organ specificity. Teratomas detected in the perinatal period mostly originate from Hensen’s node in the sacrococcygeal region or caudal pluripotent primitive stem cells [8]. Although the majority of these tumors are benign, perinatal mortality rates have been reported to be high, ranging from 13% to 16% for cases diagnosed prenatally [5]. The course of SCT diagnosed prenatally is different from SCT diagnosed postnatally. These perinatal high death rates of SCT are caused mostly by preterm labor, placentomegaly, the development of hydrops, and cardiac failure [9,10]. The evaluation of prenatal lesions, with the development of intrauterine treatment modalities in these tumors, is important for selecting suitable cases for fetal surgery.

Diagnosis is usually made during the Ultrasonography (US) scan performed in the second or third trimester of pregnancy [11,12]. On US examination, it is seen as a mass lesion with a heterogeneous echo pattern containing solid and cystic structures and may show varying degrees of blood supply [13]. There is limited data on prenatal multimodal and multiparametric imaging, of the fetus tumors [14]. Most prenatal SCTs are diagnosed in utero with US scanning. Prenatal US is the effective method for early detection and diagnosis of fetal SCT and is the primary screening method [15,16]. If an SCT is suspected on prenatal US screening, in utero fetal magnetic resonance imaging (MRI) is recommended whenever possible. MRI offers a superior anatomic resolution. Our aim in this study is to present fetal MRI findings as a single-center experience of SCT diagnosed prenatally.

## 2. Material and methods

### 2.1. Patients

The University Hospital research ethics committee approved this study (ID 2021-340). Written informed consent was waived for the retrospective analyses by the Institutional Review Board. We conducted a retrospective single-center study at our University Hospital to review all fetal SCT cases suspected before birth and confirmed postnatally between January 2011–2021. Data were collected from the digital patient archiving system (Sectra, PACS). Most cases were submitted with a presumed diagnosis of tumor or fetal hydrops. All pregnant women underwent a detailed fetal anomaly scan using high-resolution US equipment.

### 2.2. USG and MR examinations

Fetal ultrasonographic evaluations were performed by a perinatologist (at least 10 years of experience) and a radiologist (at least 15 years of experience) experienced in the obstetric US, separately. Fetal growth, exact tumor dimensions, morphology, and location, the presence or absence of additional malformations, and assessment of amniotic fluid volume were evaluated. There was no conflict between the experts in the interpretation of the images. It was agreed that MRI should be performed as advanced imaging to evaluate the extent of SCT and additional anomalies. MRI was performed via a 1.5-T magnet (Gyroscan Intera; Philips medical systems, Eindhoven, The Netherlands) with a body coil. In our center, fetal MRI (1.5 Tesla) is standard for additional evaluation. The mother was positioned either supine or in a partial left lateral decubitus position. The following imaging sequences were performed: T2-weighted Turbo Spin Echo sequences of fetus in sagittal, coronal, and axial planes (TR/effective TE, 460/80; angle of rotation, 130°; slice thickness, 4–5 mm); T1-weighted gradient-echo relative to the fetus in the axial plane (TR/TE, 11.7/4.4; angle of rotation, 65°; section thickness, 3.3 mm); and diffusion-weighted imaging in the axial plane (effective TR/TE, 4000/67; slice thickness, 3 mm). The scanning time for all was 16–18 min. All MR studies were reviewed and interpreted by two radiologists, who also knew the results of MRI results were compared with US reports and images. The presence, size, content extension, and compressive effects of each mass were determined and correlated with US findings and with postnatal studies, including surgery and pathology. MRI findings were confirmed at surgery or autopsy in all patients. Depending on the radiological findings, the extent of the SCT was classified according to the American Academy of Pediatrics, Surgical Section classification which includes four categories [17]: **Type I:** developing only outside the fetus (can have a small presacral component); the majority of cases, **Type II:** extra-fetal with intra-pelvic presacral extension, **Type III:** extra-fetal with abdominopelvic extension, **Type IV:** tumor developing entirely in the fetal pelvis. As complications, the presence of ascites and skin edema or pleural or pericardial effusion was diagnosed as hydrops. The presence of oligo- or polyhydramnios was determined by calculating the amniotic fluid index. Descriptive statistics were used due to the small number of cases. Categorical data are given as numbers (n) and percentages (%).

## 3. Results

**Figure 1:**
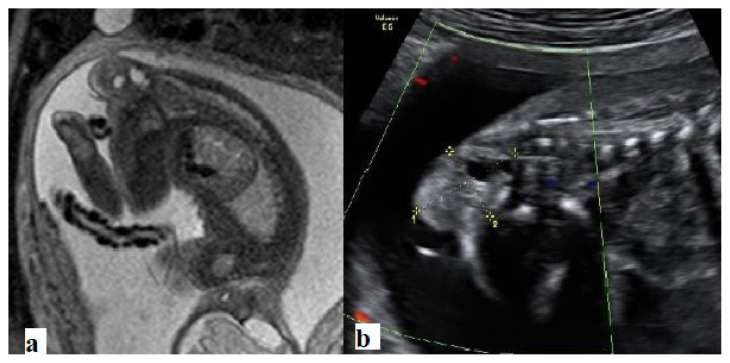
Type I sacrococcygeal teratoma. **a**. Sagittal T2-weighted, and **b**. Ultrasound images show mixed cystic and solid lesions originating from the coccyx. An intrapelvic extension is not seen.

**Figure 2:**
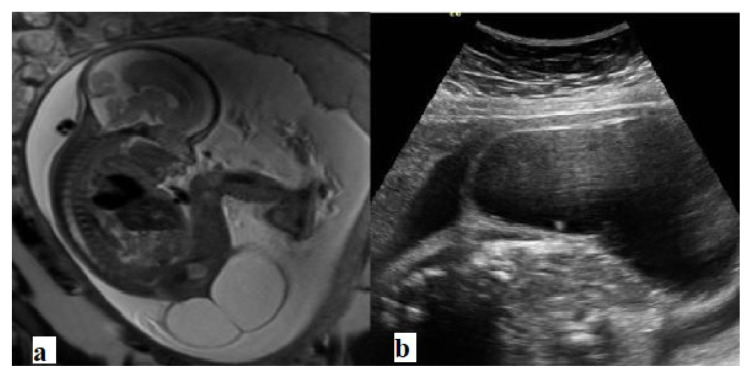
Type I sacrococcygeal teratoma. **a**. Sagittal T2 weighted, and **b**. Ultrasound images demonstrate a large, mostly cystic, mass lesion arising from the coccyx.

**Figure 3:**
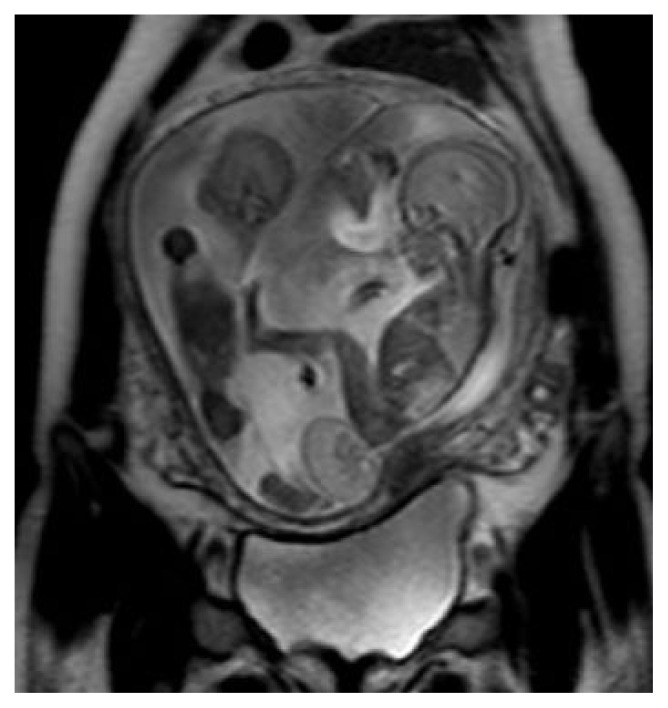
Type I sacrococcygeal teratoma. Sagittal T2 weighted image shows dichorionic-diamniotic-twins. There is a mixed solid and cystic lesion originating from the coccyx. The other fetus that had been imaged partially does not have any tumor or any anomaly.

**Figure 4:**
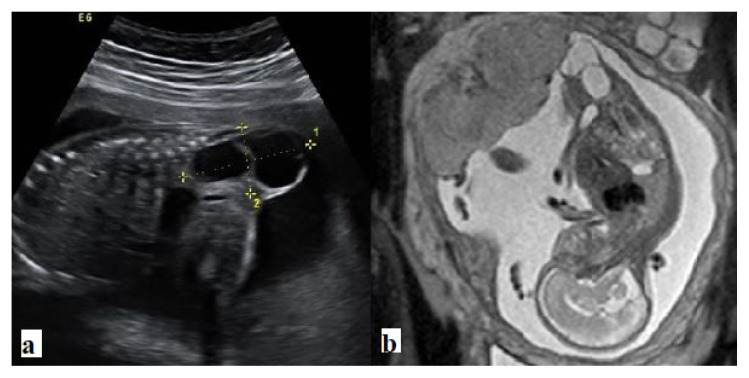
Type II sacrococcygeal teratoma. a. Ultrasound, and b. Coronal T2 weighted images show large septate cystic mass arising from coccyx with a small intrapelvic component.

**Figure 5:**
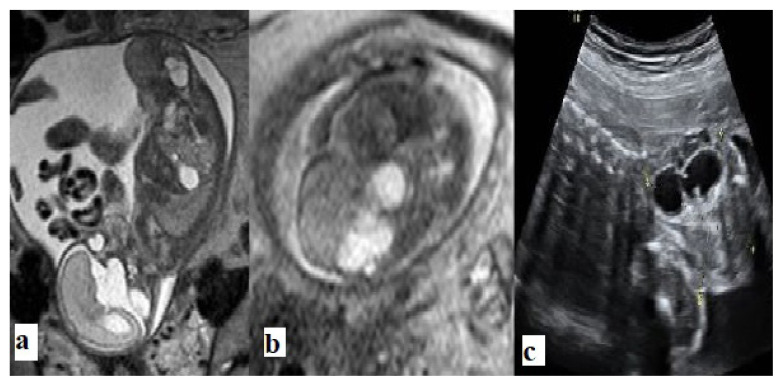
Type III sacrococcygeal teratoma. **a**. Sagittal, and **b**. Axial T2-weighted, **c**. Ultrasound images show large mixed signal intensity tumor extending into abdomen.

**Figure 6:**
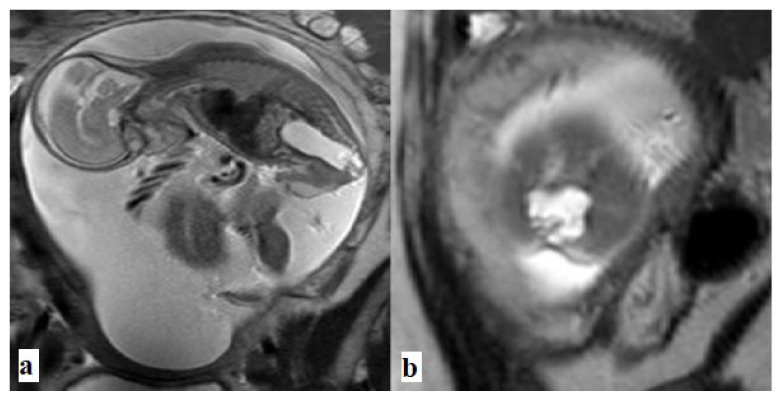
Type IV sacrococcygeal teratoma. **a**. Sagittal, and **b**. Axial T2-weighted images show tumor developed entirely in the fetal pelvis. The urinary bladder is displaced and vertical dimension is increased and contours are lobulated with increased thickness. There is also polyhydramnios.

**Figure 7:**
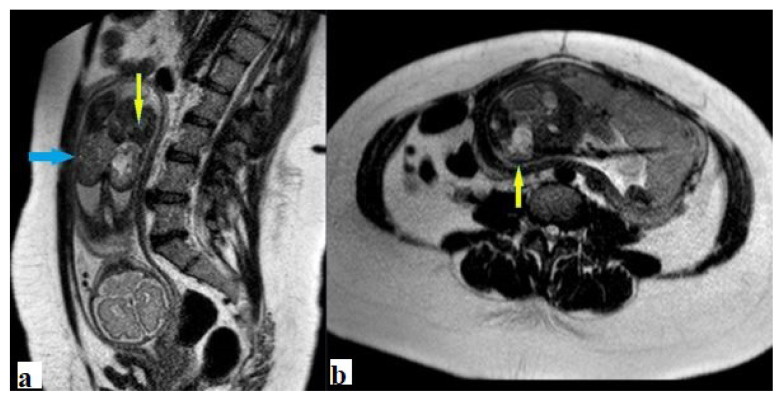
Fetus with type 1 SCT. **a**. Coronal and, **b**. axial T2 weighted images show right renal agenesis (blue arrow), left renal hydronephrosis (yellow arrow).

**Figure 8:**
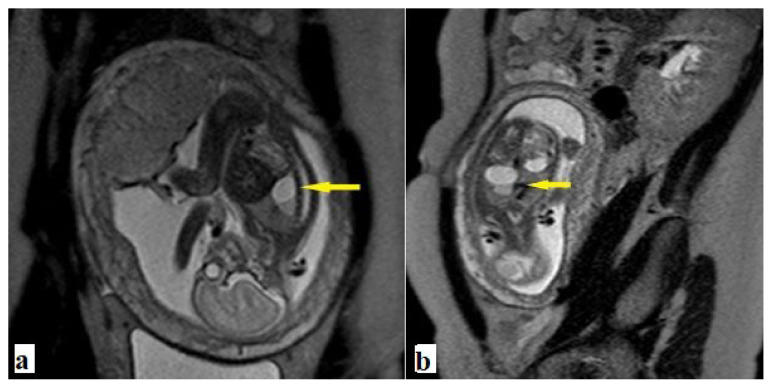
Fetus with type 2 SCT. **a**. Sagittal and, **b**. Coronal T2 weighted images show congenital airway malformation at the base of the right lung (yellow arrow).

Eleven patients diagnosed as SCT prenatally by US and further defined by MRI are included. Diagnostic quality MR images were obtained in all patients. All images taken in MR examination were evaluated. US findings are strongly correlated with MRI findings. An agreement on the extent of each mass was observed in eight patients, disagreement in one fetus was an extension of the tumor within the spinal canal recognized only at MR, and assessment of intrapelvic-abdominal extension was superior in MRI. On MRI, sacrococcygeal teratomas (n = 11) were completely cystic in four fetuses, mixed and solid in seven (Table). According to Altmand classification, there were n = 6 type I (Figures 1a, 1b, 2a, 2b, and 3), n = 2 type II (Figures 4a, 4b), n = 1 type III (Figures 5a, 5b, 5c) and, n = 2 type IV tumors (Figures 6a, 6b). MRI was superior to US for detecting displacement of the colon (n = 3), intrapelvic-intraabdominal extension (n = 1), urinary tract complication (n = 2), and intraspinal extension (n = 1). Three patients (bilateral n = 2, unilateral n = 1) had pelvicaliectasis in the kidneys on MRI. However, one of these three patients had unilateral renal ectasia, the other kidney was agenetic (Figures 7a, 7b) and there was severe oligohydramnios. In MRI, tumor compression of adjacent pelvic structures could be evaluated. However, pelvic bones and vertebral column were a limitation in evaluating the extent of SCT on sonography. Chiari type 2 malformation was present in one case (9%) of 11 fetuses and type 3 congenital pulmonary airway malformation in other cases (Figures 8a, 8b). These findings observed in MRI were also detected in the sonographic examination.

SCT causes displacement of the bladder by the effect of pelvic mass, hydronephrosis, and large convoluted ureters. The bladder displacement was observed in 3 of 11 fetuses (27.2%), and bilateral hydronephrosis in 2 fetus and unilateral hydronephrosis in 1 fetus on MRI, but only hydronephrosis was detected by sonography. In the patient with unilateral hydronephrosis, no other kidney was observed (unilateral renal agenesis), and oligohydramnios was present. In the pathological examination, the cases were documented as mature (n = 8), immature (n = 2) and mixed teratomas (n = 1).

## 4. Discussion

In this study, we aimed to evaluate imaging findings and analyze which imaging approaches are most valuable in fetuses diagnosed with prenatal SCT. SCTs are the most common tumors of the fetus and the neonate, carrying a variable prognosis. The majority tend to be benign (~80%). Alpha-fetoprotein (AFP) and beta HCG may be elevated. A pathology-based classification is as: benign (mature) 70%–80% or malignant (immature). Those presenting in older infants tend to have a higher malignant potential and those presenting in utero have a poor prognosis due to complications. It has been reported that fetal mortality is higher if the gestational week at the time of diagnosis is early in SCT [18]. Mass size, the solid component, and the vascularity of the mass are reported to be more important on prognosis than the gestational age at the time of diagnosis [18]. The prognosis of SCTs detected in the prenatal period is to be worse than those detected in the neonatal period which may be due to the fact that larger tumors are more likely to be detected in fetal life, and tumors detected in early pregnancy have greater growth potential [19]. Besides the prognosis of prenatally detected SCT is also related to its content. Predominantly solid and highly vascularized masses demonstrate a worse prognosis than tumors that are mainly avascular and cystic in appearance [20].

Complications include urologic complications (the most common cause of morbidity), high output cardiac failure from AV shunting: which in turn can cause hydrops fetalis, gastrointestinal tract obstruction, compression of underlying nerves leading to urinary/fecal incontinence, anemia, dystocia, tumor rupture. Tumor compression of the bladder outlet caused retention in the urinary system, followed by renal impairment, oligohydramnios, and pulmonary hypoplasia [12], also tumor compression or infiltration of the sacral nerves and intraspinal extension of the tumor.

Differential diagnoses include sacral chordoma, sacral meningocele, terminal myelocystocoele, and enteric (tailgut) cyst for cystic types and for type IV lesions also low-lying neuroblastoma, rhabdomyosarcoma, small round blue cell tumor in the sacral region. Treatment is with surgical excision inclusive of coccygectomy with additional chemotherapy for malignant ones.

MRI is widely used in these fetuses, although there are few studies in the literature that it is effective [14,21–23]. Majorly, the US operator-dependent technique is affected by the thickness of the abdominal fat tissue, fetal position, and amniotic fluid volume. In addition, it has limited soft-tissue contrast and decreased visual field compared with that of MRI. On the other hand, MRI has a wide window of vision unaffected by fetal position or insufficient amniotic. It can also provide a better resolution of the tumor tissue and the relationship with the surrounding tissues. After a prenatal lesion was suspected by US, fetal magnetic imaging was used to confirm the diagnosis and evaluate additional anomalies. In terms of fetal imaging, we found that MRI was superior to US in predicting tumor morphology in teratomas. More data are needed to support this finding, but preliminary results from our series show a high correlation between fetal MRI findings, outcome, and histopathology in SCTs. Two parents decided to terminate their pregnancies. Like US, MRI examination is a safe method and does not expose the patient to unnecessary risks [16,24]. The possibility of multiplanar examination and soft-tissue resolution for multisystemic evaluation in the diagnosis of fetal malformations are very helpful.

The limitation of our study is that it was designed retrospectively, US were performed by a perinatologist and a radiologist, separately. Although there was no conflict among our researchers, perhaps consensus would be a better alternative.

## 5. Conclusion

MRI is found to be superior to US, especially in the assessment of intrapelvic and intraspinal extension of the tumor, colonic displacement, and complications. While MRI contributes to the diagnosis, it also serves as a guide for treatment, birth planning, and counseling. In order to optimize pre and postnatal management for the evaluation of fetal sacrococcygeal teratoma, fetuses with SCT detected by US should be referred to MRI to evaluate tumor size, content, and extent.

## Figures and Tables

**Table:** Clinical characteristics and outcomes in 11 fetuses with SCT.

Patient	Gestational week (GW)	Tumor type	Tumor size (cm^3^)	US evidence for decompensation	Additional findings
I	21+2	1	15.9	No	
II	23+1	2	33.3	Increased AF	
III	22+1	2	27.7	No	CPAM
IV	21+4	4	25	Increased AF	
V	26+8	1	14.5	Decreased AF	Renal Agenesis
VI	19	1	52.8	No	
VII	22+5	1	150	No	
VIII	22+3	1	8	No	
IX	21+5	1	9.5	Increased AF	
X	32	3	66.5	No	
XI	18+5	4	23.5	No	Chiari Type 2

US: Ultrasonography, AF: Amniotic fluid, CPAM: Congenital pulmonary airway malformation

## References

[1] Brace V Grant SR Brackley KJ Kilby MD Whittle J Prenatal diagnosis and outcome in sacrococcygeal teratoma: a review of cases between 1992 and 1998 Prenatal Diagnosis 2000 20 51 55 10.1002/(SICI)1097-0223(200001)20:1<51::aid-pd755>3.0.co;2-o 10701852

[2] Avni FE Guibaud L Robert Y Segers V Ziereisen F MR imaging of sacrococcygeal teratoma: Diagnosis and assessment American Journal of Roentgenology 2002 178 179 183 10.2214/ajr.178.1.1780179 11756117

[3] Woodward PJ Sohaey R Kennedy A Koeller KK From the archives of the AFIP: A comprehensive review of fetal tumors with pathologic correlation RadioGraphics 2005 25 1 215 242 10.1148/rg.251045156 15653597

[4] Hambraeus M Arnbjörnsson E Börjesson A Salvesen K Hagander L Sacrococcygeal teratoma: A population-based study of incidence and prenatal prognostic factors Journal of Pediatric Surgery 2016 51 3 481 485 10.1016/j.jpedsurg.2015.09.007 26454470

[5] Swamy R Embleton N Hale J Sacrococcygeal teratoma over two decades: Birth prevalence, prenatal diagnosis and clinical outcomes Prenatal Diagnosis 2008 28 11 1048 1051 10.1002/pd.2122 18973151

[6] Flake AW The fetus with sacrococcygeal teratoma Harrison MR Evans MI Adzick NS Holzgreve W The unborn patient 3rd ed Philadelphia, PA, USA WB Saunders 2000 315 323

[7] Phi JH Sacrococcygeal teratoma: A tumor at the center of embryogenesis Journal of Korean Neurosurgical Society 2021 64 3 406 413 10.3340/jkns.2021.0015 33906346 PMC8128526

[8] Arısoy R Erdoğdu E Kumru P Demirci O Ergin N Prenatal diagnosis and outcomes of fetal teratomas Journal of Clinical Ultrasound 2016 44 118 125 10.1002/jcu.22310 26426797

[9] Kremer ME Wellens LM Derikx JP van Baren R Heij HA Hemorrhage is the most common cause of neonatal mortality in patients with sacrococcygeal teratoma Journal of Pediatric Surgery 2016 51 1826 1829 10.1016/j.jpedsurg.2016.07.005 27502009

[10] Bond SJ Harrison MR Schmidt KG Silverman NH Flake AW Death due to high-output cardiac failure in fetal sacrococcygeal teratoma Journal of Pediatric Surgery 1990 25 1287 1291 10.1016/0022-3468(90)90535-h 2286911

[11] Özkan ZS Çilgin H Aygün HB Deveci D Şimşek M Our clinical experience about prenatal diagnosis and neonatal outcomes of fetal central nervous system anomalies Journal of Maternal-Fetal and Neonatal Medicine 2011 24 3 502 505 10.3109/14767058.2010.501125 20807159

[12] Aboughalia H Noda S Chapman T Revzin MV Deutsch GH Multimodality imaging evaluation of fetal spine anomalies with postnatal correlation Radiographics 2021 41 7 2176 2192 10.1148/rg.2021210066 34723699

[13] Cass DL Fetal abdominal tumors and cysts Translational Pediatrics 2021 10 5 1530 1541 10.21037/tp-20-440 34189111 PMC8192983

[14] Ulm B Muin D Scharrer A Prayer D Dovjak G Prenatal ultrasound and magnetic resonance evaluation and fetal outcome in high-risk fetal tumors: A retrospective single-center cohort study over 20 years Acta Obstetricia et Gynecologica Scandinavica 2020 99 11 1534 1545 10.1111/aogs.13933 32525215 PMC7689914

[15] Kashyap N Pradhan M Singh N Yadav S Early detection of fetal malformation, a long distance yet to cover! Present status and potential of first trimester ultrasonography in detection of fetal congenital malformation in a developing country: Experience at a tertiary care centre in ındia Journal of pregnancy 2015 2015 623059 10.1155/2015/623059 26759727 PMC4670877

[16] Eyüboğlu İ Dinç G Fetal US and MRI in detection of craniospinal anomalies with postnatal correlation: single-center experience Turkish Journal of Medical Sciences 2021 51 3 1211 1219 10.3906/sag-2011-122 33517612 PMC8283491

[17] Altman RP Randolph JG Lilly JR Sacrococcygeal teratoma: American academy of pediatrics surgical section survey1973 Journal of Pediatric Surgery 1974 9 3 389 398 10.1016/s0022-3468(74)80297-6 4843993

[18] Zheng XQ Yan JY Xu RL Wang XC Chen X A clinical analysis of the diagnosis and treatment of fetal sacrococcygeal teratomas Cancer Management and Research 2020 12 13185 13193 10.2147/cmar.S287682 33380826 PMC7767721

[19] Akinkuotu AC Coleman A Shue E Sheikh F Hirose S Predictors of poor prognosis in prenatally diagnosed sacrococcygeal teratoma: A multiinstitutional review Journal of Pediatric Surgery 2015 50 5 771 774 10.1016/j.jpedsurg.2015.02.034 25783370

[20] Muthee BW Bray HJ Approach to the postnatal sonographic evaluation of prenatally detected abdominopelvic cysts Ultrasonography 2022 41 1 53 73 10.14366/usg.21070 34344138 PMC8696132

[21] Danzer E Hubbard AM Hedrick HL Johnson MP Wilson RD Diagnosis and characterization of fetal sacrococcygeal teratoma with prenatal MRI American Journal of Roentgenology 2006 187 4 W350 W356 10.2214/AJR.05.0152 16985105

[22] Simonini C Strizek B Berg C Gembruch U Mueller A Fetal teratomas - A retrospective observational single-center study Prenatal Diagnosis 2021 41 3 301 307 10.1002/pd.5872 33242216

[23] Zhang L Yang XH Zhao S Chen X Huang Y Combined application of prenatal ultrasound and magnetic resonance imaging in diagnosis of fetal teratoma Chinese Journal of Ultrasound in Medicine 2017 33 10 923 925 10.3877/cma.j.issn.1672-6448.2018.02.009

[24] Hubbard AM Harty MP MRI for the assessment of the malformed fetus Baillieres Clinical Obstetrics and Gynecology 2000 14 629 650 10.1053/beog.1999.0101 10985935

